# Supplementary material for: Ca2+-Induced Mitochondrial ROS Regulate the Early Embryonic Cell Cycle
Source: Cell Rep. 2018 Jan 2;22(1):218–31. doi: 10.1016/j.celrep.2017.12.042 (PMC5770342; doi:10.1016/j.celrep.2017.12.042)
Supplement: Document S1. Supplemental Experimental Procedures and Figures S1–S3 [file mmc1.pdf]

Cell Reports, Volume *22*

Supplemental Information

Ca^{2+}-Induced Mitochondrial ROS Regulate the Early Embryonic Cell Cycle

Yue Han, Shoko Ishibashi, Javier Iglesias-Gonzalez, Yaoyao Chen, Nick R. Love, and Enrique Amaya

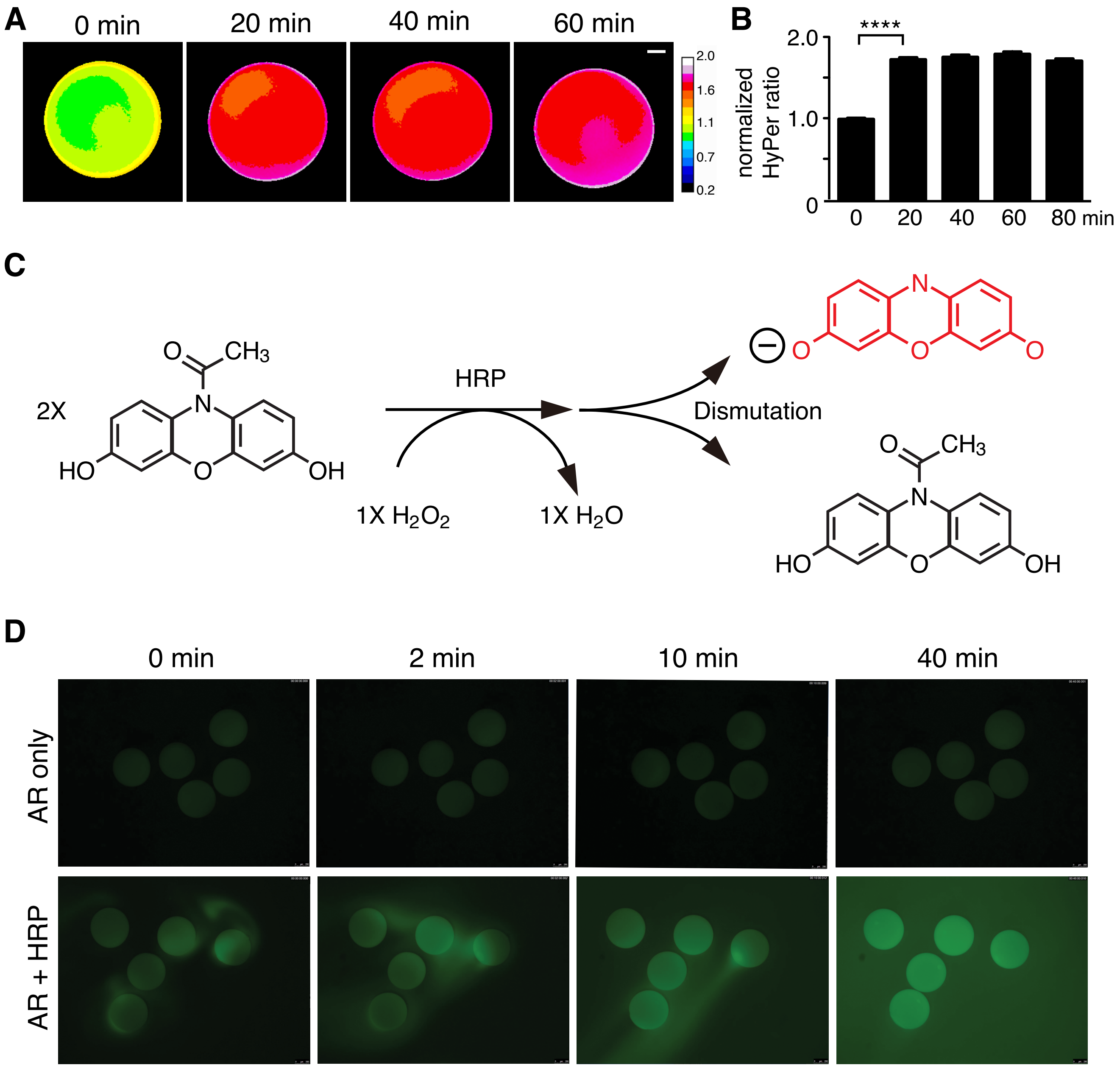

Figure S1. Related to Figure 1. ROS are generated in oocytes after prick activation. (A) Oocytes obtained from a transgenic female expressing HyPer were prick-activated and imaged every 20 min. (B) Quantification of HyPer ratio in A. n = 60. ****p < 0.0001, 20 min compared to t0, paired t-test. Error bars represent mean ± SEM. (C) Amplex Red (AR) gives rise to fluorescent resorufin reacting specifically with H_2O_2 in the presence of horseradish peroxidase (HRP). (D) 10 nl of 10 mM AR was injected into albino mature oocytes with or without 320 units/ml HRP. The strong fluorescence (pseudo color) was observed just after AR injection with HRP. These pictures are from a representative of at least three independent experiments. See also Movie S2.

A

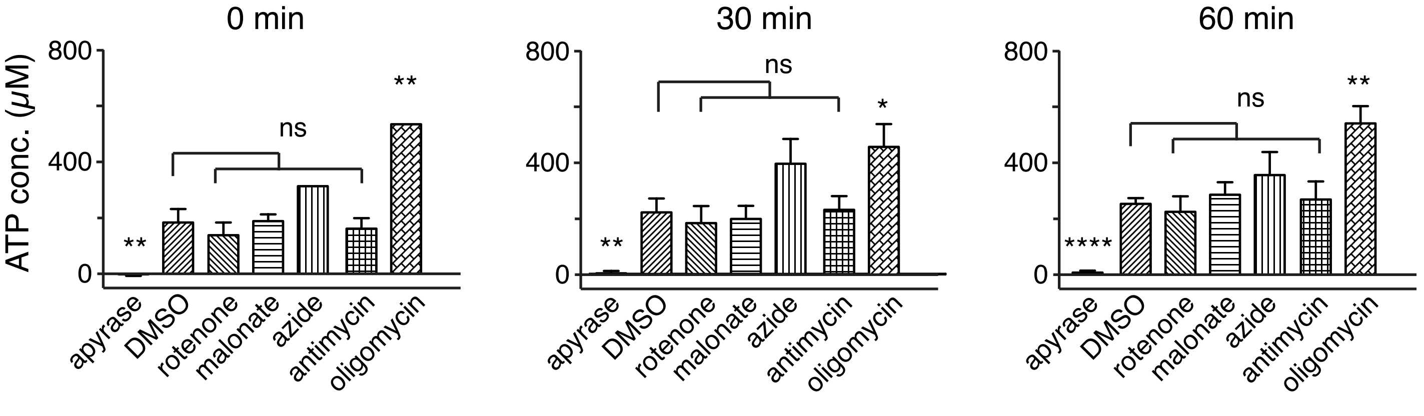

B

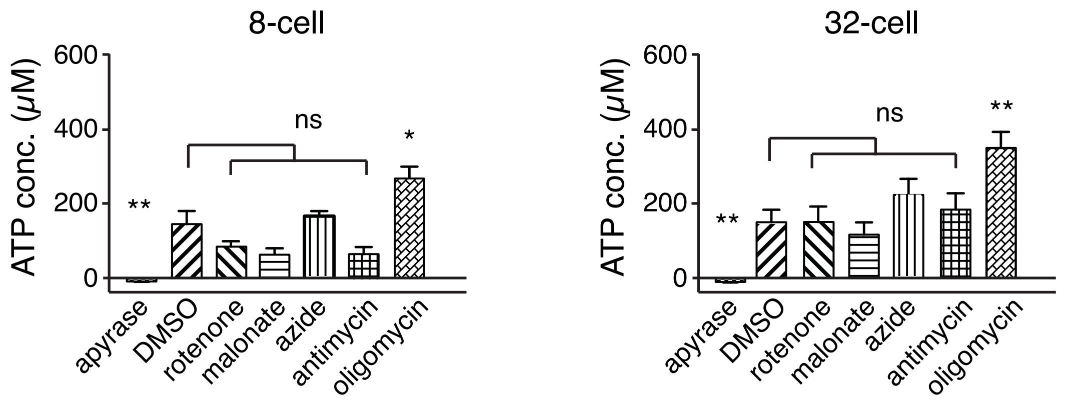

Figure S2. Related to Figures 3 and 5. ATP levels are not affected by mtROS inhibitors. (A) The amount of ATP was measured at 0, 30 and 60 min after prick-activation in the oocytes treated with mitochondrial inhibitors. As a negative control, the extract was treated with apyrase ATPase. Three independent experiments were performed with technical duplicates. Error bars represent mean ± SEM. *$p < 0.05$, **$p < 0.01$, ****$p < 0.0001$; ns, not significant, compared to DMSO control, Unpaired t-test. For apyrase at 0 min and 30 min and azide at 60 min, Mann-Whitney test was used. (B) The amount of ATP in the embryos treated with mitochondrial inhibitors was measured at the 8-cell and 32-cell stages. There was no significant decrease of ATP by inhibitor treatment. Six independent experiments were performed with technical duplicates. Error bars represent mean ± SEM. *$p < 0.05$, **$p < 0.01$; ns, not significant, compared to DMSO control, Unpaired t-test. For apyrase, Mann-Whitney test was used.

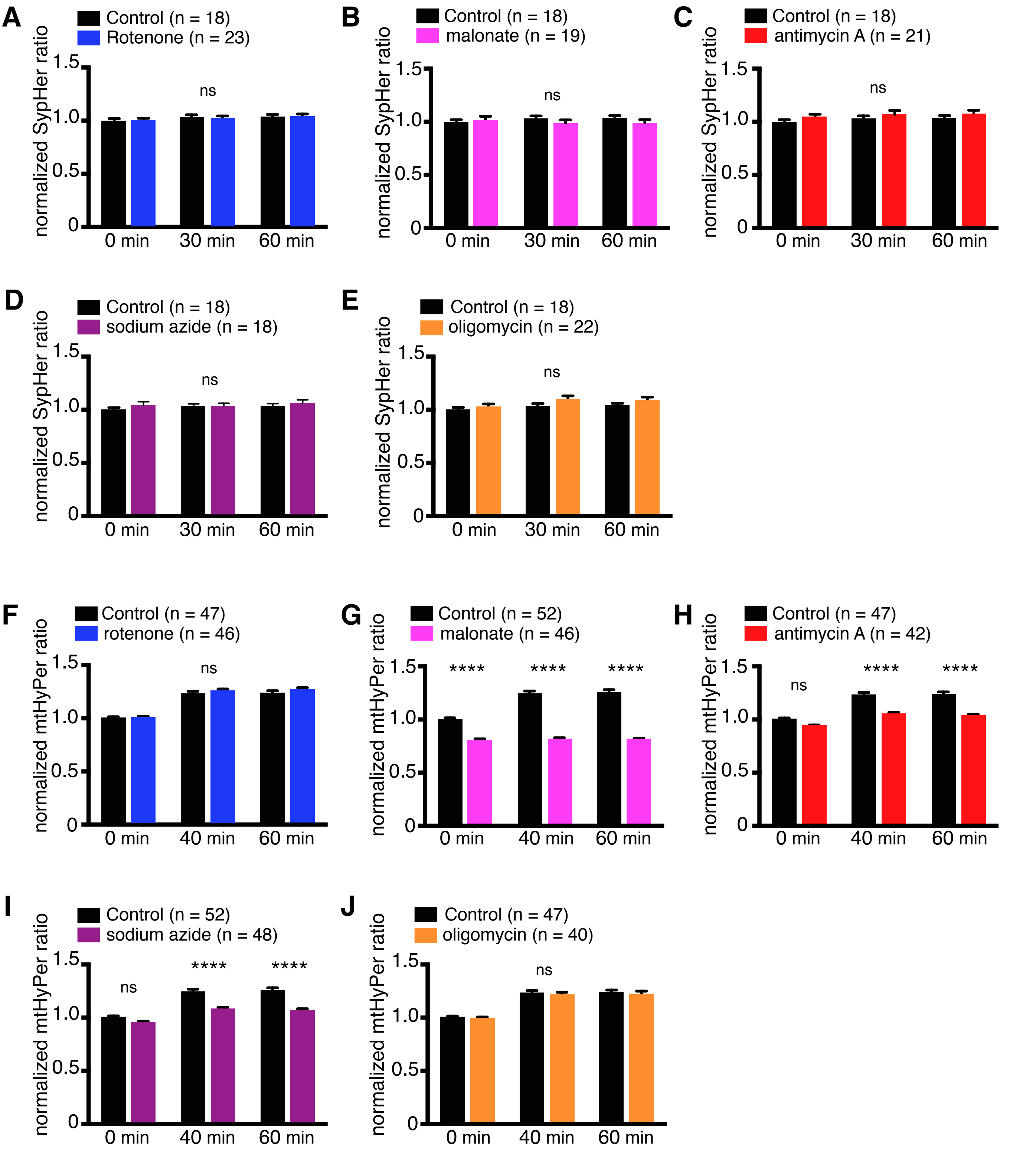

Figure S3. Related to Figure 3. SypHer ratio in mature oocytes treated with mitochondrial inhibitors (A-E) and decreased ROS production detected by mitoHyPer in mature oocytes treated with mitochondrial inhibitors (F-J).
(A-E) Quantification of normalized SypHer ratio of oocytes treated with 1 µM rotenone (A), 10 mM malonate (B), 10 µM antimycin A (C), 3 mM sodium azide (D) and 6 µM oligomycin. No significant change of SypHer ratio was observed (two-way ANOVA). (F-J) Quantification of normalized mitoHyPer ratio of oocytes treated with 1 µM rotenone, no significant decrease in ratio was found (A), 5 mM malonate, 19.8% at 0 min, 34.6% at 40 min and 35.7% at 60 min decrease in ratio (B), 10 µM antimycin A, 14.5% at 40 min and 16.5% at 60 min decrease in ratio (C), 1 mM sodium azide, 13.1% at 40 min and 14.9% at 60 min decrease in ratio (D) and 6 µM oligomycin, no significant decrease in ratio was found (E). Error bars represent mean ± SEM. ****$p < 0.0001$; ns, not significant. two-way ANOVA and Sidak post hoc tests.

Supplemental Experimental Procedures

Resource Table

REAGENT or RESOURCE	SOURCE	IDENTIFIER
**Antibodies**		
Mouse monoclonal Anti-α-Tubulin	Sigma	T9026
Goat anti-Mouse IgG (Fab specific)–TRITC	Sigma	T7782
Rabbit polyclonal anti-Lamin B1	Abcam	ab16048
Goat anti-Rabbit IgG (H+L) Alexa Fluor 647	Invitrogen	A21244
Mouse monoclonal anti-*Xenopus* Cdc25C	Dr. J. Gannon	Clone DG6
Mouse monoclonal anti-Xenopus Cyclin B2, [X29.2]	Abcam	ab18250
CDC2 (p34) Mouse Monoclonal Antibody (clone A17)	Invitrogen	33-1800
Phospho-cdc2 (Tyr15) Antibody	Cell Signaling	#9111
Rabbit monoclonal anti-Cdc25C	Abcam	ab32444
Rabbit monoclonal Anti-Cdc25C (phospho S216)	Abcam	ab32051
Goat polyclonal anti-Rabbit Immunoglobulins HRP	Dako	P0448
Goat polyclonal anti-Mouse Immunoglobulins HRP	Dako	P0447
**Chemicals, Peptides, and Recombinant Proteins**		
Progesterone	Sigma	P0130
Calcium Ionophore A23187	Sigma	C7522
Diphenyleneiodonium, DPI	Sigma	D2926
apocynin	Sigma	W508454
Rotenone	Sigma	R8875
Diethyl malonate	Sigma	D97754
Sodium azide	Sigma	08591
Antimycin A	Sigma	A8674
Oligomycin	Sigma	O4876
Ruthenium red	Sigma	R2751
Apyrase	Sigma	A6535
Amplex red	Invitrogen	A12222
Horse radish	Sigma	P8375
Sytox Green	Invitrogen	S34860

Carbonyl cyanide 3-chlorophenylhydrazone, CCCP	Sigma	C2759
**Critical Commercial Assays**		
ATP Determination Kit	Invitrogen	A22066
Passive Lysis Buffer	Promega	E1941
**Experimental Models: Organisms/Strains**		
Xenopus laevis	EXRC	Wild-type
Xenopus laevis, CMV-hyper line	(Love et al., 2013)	EXRC #55 and #63
Xenopus laevis, CMV-YFP line	this study	N/A
**Recombinant DNA**		
pCS2+ HyPer	(Love et al., 2013)	N/A
pCS2+ mitoHyPer	this study	N/A
pCS2+ R-GECO	K. Dorey	N/A
pCS2+ CDC25C	this study	N/A
pCS2+ CDC25C C330S C377S	this study	N/A
pCS2+ Xt-ccdc109b	this study	N/A
pCS107 Xt-inpp5a	(Gilchrist et al., 2004)	Tegg120m23
**Sequence-Based Reagents**		
mcub Forward 5'-ATGCCTGCTCTCGTCGG-3'	Sigma	N/A
mcub Reverse 5'-TTCTTTTTCGTTCAGCTGTTC-3'	Sigma	N/A
**Software and Algorithms**		
ImageJ	NIH	N/A
Metamorph image acquisition software	Molecular Devices	N/A
Prism	GraphPad	http://www.graphpad.com/
**Other**		
SP6 Message Machine	Invitrogen	AM1340

CONTACT FOR REAGENT AND RESOURCE SHARING

Further information and requests for resources and reagents should be directed to the lead contact, Enrique Amaya (enrique.amaya@manchester.ac.uk).

PLASMIDS CONSTRUCTION

MitoHyPer and SypHer constructs were gifts from Dr. V. Belousov. The MitoHyPer ORF obtained by digesting with *Not*I followed by Klenow and *Nhe*I, and SypHer ORF obtained by digesting with *Hind*III followed by Klenow and *Nhe*I was subcloned into the pCS2+ vector. *X. tropicalis mcub* (*ccdc109b*) was cloned by RT-PCR. Human CDC25C wild type and cysteine mutant obtained from Addgene (#10964 and #10965 respectively) and ORFs obtained by digesting with *Nco*I and *Xba*I were subcloned into pCS2+ vector.

CA2+ AND HYPER IMAGING

Oocytes were injected with 20 ng of R-GECO RNA, matured and imaged. Images were captured via a Nikon A1 microscope. The settings were as follows, pinholes [46 μm], scan selection [Galvano], format [512 x 512]. Images DsRed were excited with the 543 nm laser lines. For laser wound, laser was set as follows: wavelength [561 nm], laser power [40], pulse [10]. Embryos from transgenic female expressing HyPer or oocytes injected with HyPer RNA were imaged using a Nikon TE2000 PFS microscope. Signals were excited with filters of BP430/24 and BP500/20, and detected with a filter of BP535/30 unless otherwise indicated. Images were analyzed by subtracting background, smoothing, formatting to 32-bit, then dividing using ImageJ. Since the HyPer ratio before activation vary among the different batches of oocytes, the ratio for control before activation was set as 1.0 and used for normalization for all samples.

DETECTION OF ROS BY AMPLEX RED IN OOCYTES

Albino oocytes were injected with 10 nl of solution containing 10 mM Amplex Red with or without 320 units/ml HRP. Time lapse movies were taken every 1 min after injection using Leica M205FA with DSR filter and the camera, DFC365FX.

MEASUREMENT OF ATP

ATP was measured using the ATP-determination Kit (Invitrogen) according to the manufacturer's protocol. *Xenopus laevis* eggs were collected and immediately lysed with ice-cold 1X passive lysis buffer (Promega) and centrifuged at 13500 rpm for 10 min at 4 ˚C. A 10 μL of the supernatant was diluted in 990 μl water. Then 10 μl of sample or 10 μL ATP standard solution was added to 90 μL of reaction buffer in each well of a 96-well plate. Luminescence was measured using Mithras LB 940 Multimode Microplate Reader. 1 unit/mL apyrase was employed in this assay as a negative control. All experiments were run in biological triplicates and technical duplicates, and the background luminescence was

subtracted from the measurement. ATP concentrations in experimental samples were calculated from the ATP standard curve.

IMMUNOBLOTTING

6-10 embryos were frozen in dry ice, kept at -80 ˚C until use, and homogenized in the extraction buffer (100 mM NaCl, 50 mM Tris, 5 mM EDTA, 1% NP-40, pH 7.5) containing cOmplete Mini EDTA-free protease inhibitor (04693159001, Roche) and PhosSTOP phosphatase inhibitor (04906845001, Roche) and centrifuged at 13000 rpm for 5 min. The supernatant was dissolved in Laemmli's sample buffer containing 5% β-mercaptoethanol and heated at 95˚C for 5 min. Aliquots equivalent to 1-2 oocytes or embryos were subjected to 7.5% or 10% SDS-PAGE. Proteins on the gel were transferred to PVDF membrane and incubated with antibodies. The following antibodies were used for western blot analyses: anti-*Xenopus* Cdc25C mouse monoclonal (hybridoma, clone DG6 is gift from Dr J. Gannon, The Francis Click Institute, supernatant 1:500 dilution), anti-Xenopus Cyclin B2 mouse monoclonal (1:1000 dilution), anti-Phospho-cdc2 (Tyr15) (1:1000 dilution), anti-CDC2 (p34) Mouse monoclonal (1:1000 dilution), anti-Cdc25C (1:1000 dilution), anti-Cdc25C phospho S216 (1:1000 dilution), anti-α-tubulin (1:50000 dilution), anti-rabbit IgG/HRP (1:25000 dilution), and anti-mouse IgG/HRP (1: 25000 dilution). The signal was visualized using chemiluminescence (Immobilon, Millipore).

IMMUNOHISTOCHEMISTRY

Whole mount immunohistochemistry was carried out according to (Chalmers et al., 2003) with a modification in which pigmented embryos were bleached in 10% H_2O_2/PBS for 1 hour after fixation. The following antibodies were used: anti-Lamin B1 (1:500) with anti-rabbit Alexa Fluor 647 (1:500) and anti-α-Tubulin (1:2000) with anti-mouse TRITC (1:250). SYTOX Green was used to stain DNA (1:100). Cleared embryos with Murray's were mounted inside of the ring made of vacuum grease (Dow Corning® high-vacuum silicone grease, Sigma, Z273554) on a coverslip, and another coverslip was put on the top with a slight pressure. Confocal images were acquired by FV1000 (Olympus) using laser 488 and 543 with filter sets for Acridine Orange, TRITC and Alexa 647.

NUCLEAR TRANSPLANTATION FOLLOWED BY HYPER/SYPHER IMAGING

Oocytes injected with 20 ng HyPer or SypHer RNA were matured by adding progesterone and injected with sperm nuclei as described in (Amaya and Kroll, 1999). Successfully dividing embryos were imaged every 30 seconds for 3-4 hours using filter sets described above.

SUPPLEMENTAL REFERENCES

Amaya, E., Kroll, K.L., 1999. A method for generating transgenic frog embryos. Methods Mol. Biol. 97, 393–414. doi:10.1385/1-59259-270-8:393

Chalmers, A.D., Strauss, B., Papalopulu, N., 2003. Oriented cell divisions asymmetrically segregate aPKC and generate cell fate diversity in the early *Xenopus* embryo. Development 130, 2657–2668.

Gilchrist, M.J., Zorn, A.M., Voigt, J., Smith, J.C., Papalopulu, N., Amaya, E., 2004. Defining a large set of full-length clones from a *Xenopus tropicalis* EST project. Dev Biol 271, 498–516. doi:10.1016/j.ydbio.2004.04.023

Love, N.R., Chen, Y., Ishibashi, S., Kritsiligkou, P., Lea, R., Koh, Y., Gallop, J.L., Dorey, K., Amaya, E., 2013. Amputation-induced reactive oxygen species are required for successful *Xenopus* tadpole tail regeneration. Nat. Cell Biol. 15, 222–228. doi:10.1038/ncb2659
